# Triclosan-resistant small-colony variants of *Staphylococcus aureus* produce less capsule, less phenol-soluble modulins, and are attenuated in a *Galleria mellonella* model of infection

**DOI:** 10.1099/mic.0.001277

**Published:** 2023-01-30

**Authors:** Dina Altwiley, Tarcisio Brignoli, Seána Duggan, Ruth C. Massey

**Affiliations:** ^1^​ School of Cellular and Molecular Medicine, University of Bristol, Bristol, UK; ^2^​ Department of Biological Sciences, University of Jeddah, Jeddah, Saudi Arabia; ^3^​ Dipartimento di Bioscienze, Università degli studi di Milano, Milan, Italy; ^4^​ MRC Centre for Medical Mycology, University of Exeter, Exeter, UK; ^5^​ Schools of Microbiology and Medicine, and the APC Microbiome Ireland, UCC, Cork, Ireland

**Keywords:** *Staphylococcus aureus* capsule SCV

## Abstract

In recent work we identified genes that confer the slow-growing and antibiotic-resistant small-colony variant (SCV) form of *Staphylococcus aureus *, as associated with the amount of capsule the bacteria produce. In this study we isolated a triclosan-resistant SCV (tr-SCV) and demonstrated that it produces significantly less capsule, an effect that appears to be mediated at the transcriptional stage. As with other SCVs, we found that the tr-SCV produces less toxins, and when compared to both a capsule and an Agr mutant we found the tr-SCV to be significantly attenuated in an insect model of infection.

## Background

*Staphylococcus aureus* is an opportunistic human pathogen that causes a broad spectrum of infections, ranging from relatively minor superficial skin infections to life-threatening diseases such as osteomyelitis, endocarditis and bacteraemia [1]. This pathogen’s ability to cause such infections is dependent on its many virulence factors, which include surface-associated adhesins, a capsular polysaccharide, exoenzymes and toxins, as well as their coordinated regulation and associated metabolism [1–4]. Capsular polysaccharide (CP) is one of the most important virulence factors [5], which contributes to the ability of the bacteria to avoid and survive phagocytosis, and is critical to their ability to cause disease [5–9].

In addition to their virulence and immune evasion capacity, antimicrobial resistance is a major problem that affects our ability to treat *S. aureus* infections. Not only can they acquire resistance mechanisms horizontally, such as the emergence of methicillin-resistant *S. aureus* (MRSA) strains [10], but they can also transiently switch between high and low levels of resistance, as seen in the case of small-colony variants (SCVs) [11]. *S. aureus* SCVs are slow-growing subpopulations that can be recovered from patients with persistent or relapsing infections [11, 12], especially those being treated with aminoglycoside antibiotics [11–13]. SCVs are characterized by the formation of pinpoint colonies on agar due to their slow growth rate, reduced pigmentation and increased resistance to specific antimicrobial agents [14]. These growth defects are as a result of mutations in several biochemical pathways that result in auxotrophies for growth factors such as hemin, thymidine and menadione that confer increased resistance to the antibiotic gentamicin [11–14], or an auxotroph for fatty acids that confers increased resistance to the antibacterial agent triclosan [15–19].

In recent work we identified an association between genes associated with the SCV phenotype and capsule production [20]. We functionally verified this association and demonstrated that a menadione auxotrophic SCV did not produce capsule, whereas a hemin autotropic SCV did. In this study we sought to verify how capsule production is affected in a fatty acid-auxotrophic, triclosan-resistant SCV (tr-SCV). There are several genes associated with this SCV phenotype, all part of the fatty acid synthesis type II (FASII) pathway (i.e. *fabD*, *accA*, *accB*, *accC* and *accD* [15–19]. The tr-SCV we isolated had a mutation in the *accA* gene, which encodes acetyl coenzyme A (acetyl-CoA) carboxylase, the first enzymes in this FASII pathway [21, 22]. The production of capsule by this SCV was significantly reduced, as was its production of the phenol-soluble modulins (PSMs) family of toxins. Together with its growth defect, this culminated in a *S. aureus* strain that was significantly attenuated in an insect model of infection.

## Methods

### Bacterial growth conditions and selection of the tr-SCV form of *S. aureus* strain Newman

The bacterial strains used in this study are listed in Table 1. *S. aureus* strains were grown in either tryptic soy broth (TSB) or on tryptic soy agar (TSA) overnight at 37 °C. To isolate a tr-SCV, sterile filter discs impregnated with 32 µg ml^−1^ of triclosan were applied to the surface of a TSA plate that had been inoculated with a lawn of Newman, and this plate was then incubated for 3 days at 37 °C. After 3 days, bacteria at the edge of the zone of inhibition were moved onto a fresh TSA plate containing 32 µg ml^−1^ concentration of triclosan and purified to a single colony. Where described, Tween 80 was added to the molten TSA agar at a concentration of 0.1 % and allowed to set. For the triclosan inhibition assay a sterile filter disc was soaked in a solution of 32 mg ml^−1^ triclosan and the disc plates on the surface of an inoculated TSA plate.

**Table 1.** List of strains used in this study

Strain name	Description	Reference
Newman	Wild-type *S. aureus* MSSA	[23]
Newman tr-SCV	Triclosan-resistant SCV	This work
Newman tr-SCV (pRMC2)	Triclosan-resistant SCV containing the empty pRMC2 plasmid	This work and [24]
Newman tr-SCV (paccA)	Triclosan-resistant SCV containing the accA gene cloned into pRMC2	This work
JE2 *capE::tn*	MRSA strain JE2 containing a transposon in the *capE* gene	[25]
JE2 *agrA::tn*	MRSA strain JE2 containing a transposon in the a*grA* gene	[25]
Newman *capE::tn*	Newman containing the transposon in the *capE* gene from strains JE2	This work
Newman *agrA::tn*	Newman containing the transposon in the *agrA* gene from strains JE2	This work

### DNA extraction and genome sequencing of the tr-SCV

DNA extraction and whole-genome sequencing (WGS) of Newman wild-type and Newman tr-SCV were performed by MicrobesNG using the Illumina HiSeq platform. Sequences (length 2×250 bp paired-end reads) were analysed through multiple pipelines; starting by using Kraken to identify the closest reference genome [23], which confirmed that all sequences were *S. aureus *. The data were then *de novo* assembled using SPAdes [24] followed by variant calling against the closest reference genome (Newman) [25]. The sequence accession number is PRJNA872918. Nonsynonymous mutations were identified by comparing Newman SCV to the untreated parent isolate sequence data.

### Cloning of the *accA* gene for tr-SCV complementation

To complement the tr-SCV, primers were designed to amplify the entire *accA* gene, including restriction sites for the *Kpn*I and *Sac*I restriction enzymes indicated in italics:

F: CGC*GGTACC*TTATTCTATATAAGAACCGATATTTCTG

R: CGG*GAGCTC*CTAAAAATCCATCAAGAGGTGAC

These primers were used to amplify the *accA* gene from genomic DNA from Newman wild-type as the template using high-fidelity phusion polymerase. The following PCR conditions were used: 35 cycles of 30 s initial denaturation at 95 °C, annealing at 52 °C for 10 s, an extension at 72 °C for 30 s for every 1 kb of DNA to be amplified, and a final extension of 5 min at 72 °C. The PCR products were purified using the QIAquick PCR Purification kit (Qiagen, Crawley, UK), and both the purified PCR product and the pRMC2 plasmid [26] were subjected to double digestion using *Kpn*I and *Sac*I (New England Biolabs). The digested products were cleaned using the QIAquick PCR Purification kit, ligated together using T4 DNA ligase and transformed into *E. coli* strain DH5*a*. The successful constructs were verified by restriction digest and sequencing and electroporated into *S. aureus* strain RN4220 strain, which can accept DNA from *E. coli* and modify it for acceptance into other *S. aureus* strains. The plasmid was then moved from RN4220 into the tr-SCV by electroporation.

### Construction of Newman mutants

The *capE* and *agrA* mutants of strain Newman were constructed by transducing the appropriate transposon mutant from the Nebraska library [27] into strain Newman by phage Phi 11 transduction [28]. In brief, an overnight culture of Newman was diluted 1 : 100 in 25 ml TSB in a 250 ml flask and incubated at 37 °C with shaking for 1 h, and the cells harvested by centrifugation. The cell pellet was resuspended in 0.5 ml TSB to which 40 µl of 10 mg ml^−1^ CaCl_2_ solution was added, followed by sufficient volume of Phi11 stock to result in a multiplicity of infection (m.o.i.) of 0.1. This was incubated at room temperature for 10 min and then at 30 °C for 35 min static, and an additional 2.5 ml of TSB was added and cells were harvested by centrifugation. The pellet was resuspended in 5 ml of fresh TSB and incubated for a further 1.5 h at 37 °C, and the suspension played on selective agar (TSA plus erythromycin 10 µg ml^−1^) for 24–48 h. Successful transductants were verified by their antibiotic resistance profile (i.e. oxacillin-sensitive and erythromycin-resistant) and by PCR across the transposon insertion site [27].

### Capsule immunoblotting

The production of the capsule for each strain was determined using a previously described dot immunoblot assay [20]. Each strain was grown on TSA and incubated at 37 °C for 18 h. Colonies were scraped from the plate surface and resuspended in TSB. The bacterial density for each strain suspension was adjusted to an OD_600_ of 2 and then serially diluted (twofold, down to a 1/8 dilution), and 3 µl of the dilutions were spotted on nitrocellulose membrane and allowed to dry. The membrane was washed in trypsin to remove all adherent cells and proteins. The membrane was then incubated for 1 h in a blocking buffer containing bovine serum albumin (BSA), and incubated with capsule anti-serum, followed by a secondary antibody labelled with horseradish peroxidase (HRP). Reactivity was detected via colorimetric detection using the Opti-4CN system according to the manufacturer’s instructions and digitally photographed. The reactivity of the bacterial dots to the antiserum was measured by densitometry using ImageJ [29] and paired *t*-tests were used to determine the statistical differences between the strains.

### mRNA extraction and qRT-PCR quantification of the *capE* gene

The bacteria were grown in TSB at 37 °C and incubated in a shaking incubator for 18 h, and the bacterial density of each culture was normalized to an OD of 2. Total RNA was extracted using the Quick-RNA Fungal/Bacterial Miniprep kit (Zymo Research) according to the manufacturer’s instructions. RNA integrity was checked by running 5 µl aliquot of the RNA on a 1 % agarose gel and observing the intensity of the ribosomal RNA (rRNA). RNA samples were treated with TURBO DNase (Invitrogen) to eliminate any genomic DNA contamination. To verify that the samples were free from any DNA contamination, RNA samples were subjected to quantitative reverse-transcriptase PCR (qRT-PCR) alongside a no-template control (NTC) and 2.5 ng of a known genomic DNA, and threshold rates were compared.

Complementary DNA (cDNA) was generated from the mRNA samples using the qScript cDNA Synthesis kit and following the manufacturer’s protocol (Quantabio), and the cDNA was used as a template for the qPCR reaction. The primers used are listed in Table 2 and the SensiFAST SYBR No-ROX kit (Bioline) was used. The reverse-transcriptase PCR (RT-PCR) was performed as follows: 10 µl 2× SensiFAST SYBR mix, 0.8 µl of 10 µM forward primer, 0.8 µl of 10 µM µl reverse primer, 1 µl cDNA and RNase-free water up to a total of 20 µl volume. The PCR cycles consisted of initial denaturation at 95 °C for 2 min followed by 40 cycles of denaturation at 95 °C for 10 s, annealing at 55 °C for 60 s and an extension at 72 °C for 10 s. RT-PCR was carried out in triplicate for each sample with three or more biological repeats. The ratio of the *capE* and *gyrA* transcript number was calculated according to the 2^−ΔΔCT^ method [30].

**Table 2.** List of primers used for real-time PCR

Primer	Sequence (5′−3′ end)
RT *capE* F	ACATTGGTGATGTGCGTGAT
RT *capE* R	TCACATGACGGCACTTGTTT
RT *gyrA* F	CCAGGTAAATTAGCCGATTGC
RT *gyrA* R	AAATCGCCTGCGTTCTAGAG

### PSM harvesting and analysis

The method used to harvest the PSMs was similar to one described previously [31], with the following exception: bacteria were grown overnight in 5 ml of TSB incubated at 37 °C with shaking for 18 h (180 r.p.m). After centrifuging the cultures, 4 ml of the supernatant was added to 1 ml of trichloroacetic acid (TCA) and placed on ice for 1 h. The samples were then centrifuged at 3000 r.p.m for 10 min and the pellets washed three times in ice-cold acetone. The samples were then dissolved in 100 µl of 8M urea and run on a 10 % SDS-PAGE for 90 min at 100V, mA 300, and then stained with the Gel Code Blue Stain (Thermo Fisher).

### *Galleria mellonella* infection

*G. mellonella* larvae were purchased from Live Foods Direct (Sheffield, UK) and stored at 4 °C in the dark for a maximum of 7 days. Ten larvae were inoculated with each strain as described previously [15, 32]. In brief, overnight *S. aureus* cultures were grown in TSB and washed twice in sterile phosphate-buffered saline (PBS) before being diluted to an OD_600_ of 0.6. A Hamilton 750 syringe was used to inject the haemocoel of each of the 10 larvae through left proleg with the washed bacterial suspension (10 µl, i.e. 1×10^8^ c.f.u. per larvae). Each group of larvae were placed in a sterile Petri dish and incubated at 37 °C in the dark. A control group (not injected) and a PBS-injected group were also incubated as negative controls. The survival of each group was recorded daily for 3 days, and larvae were considered dead when they appeared melanized (black) or did not respond to touch. The experiment was performed three independent times and the results are presented as a Kaplan–Meier curve and a log-rank (Mantel–Cox) test, calculated using Prism v.9.

## Results and discussion

**Fig. 1.**
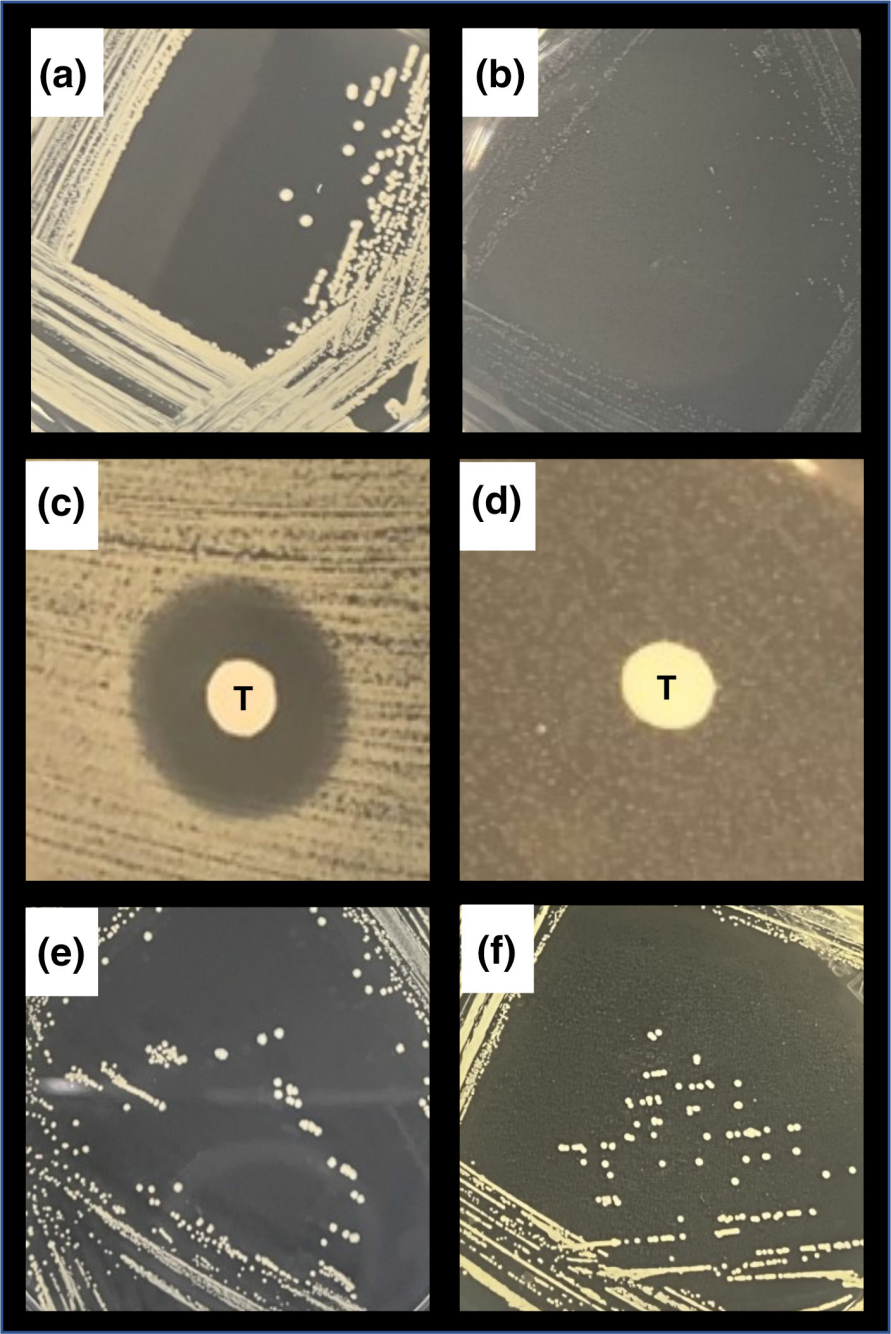
Triclosan-resistant small-colony variants of *S. aureus *. (**a**) Normal-sized colonies of wild-type *S. aureus* strain Newman. (**b**) SCVs of Newman that were selected on triclosan. (**c**) Zone of inhibition of growth of Newman by triclosan (T). (**d**) Triclosan does not inhibit the growth of the triclosan-selected SCV (T). (**e**) Tween 80 restores the growth of the triclosan-selected SCV to wild-type levels of growth. (**f**) The expression of the wild-type *aacA* gene from the pRMC2 plasmid complements the *aacA* mutation and restores growth of the tr-SCV to wild-type levels.

In previous work we identified mutations in genes involved in the triclosan-resistant SCV (tr-SCV) form of *S. aureus* as being associated with capsule production [20]. To examine this association, we isolated a tr-SCV by growing *S. aureus* strain Newman in the presence of 32 µg ml^−1^ of triclosan for 3 days. Strain Newman was used, as it is relatively easy to genetically manipulate and produces an abundant capsule for which we have antiserum. The resulting strain was further sub-cultured and streaked out for single colonies on TSA to examine the growth morphology, where the SCV form was evident (Fig. 1a, b). This SCV phenotype was stable over five successive streakings for single colonies. To confirm that the isolated SCV was triclosan-resistant we inoculated the surface a TSA plate with either the wild-type or isolated SCV form, and a sterile filter disc impregnated with triclosan (32 µg ml^−1^) was placed onto the plate and the bacteria allowed to grow. There was a large zone of growth inhibition around the triclosan disc for the wild-type strain, but none for the SCV, confirming its triclosan resistance phenotype (Fig. 1c, d). Previous work has demonstrated that Tween 80 can supplement the loss of the fatty acid biosynthesis pathway associated with the tr-SCV phenotype and restore growth back to wild-type levels. To test this, we incorporated Tween 80 (0.1 %) into TSA and demonstrated that on this medium the growth of the tr-SCV colonies was restored to wild-type levels (Fig. 1e).

To identify the mutation responsible for the tr-SCV phenotype we sequenced its genome and compared it to that of the wild-type strain. The tr-SCV had a mutation in the *accA* gene that encodes the acetyl-CoA carboxylase, the enzyme that initiates the general fatty acid synthase type II (FASII) cycle, which is consistent with other studies of tr-SCVs [18, 19]. We did not observe any other mutations associated with this SCV phenotype. The mutation was a G-to-A nucleotide change at position 587 in the *accA* gene, which confers a G197D change to the protein sequence. To verify the role of this enzyme in this tr-SCV phenotype we cloned the wild-type version of the gene into the pRMC2 expression vector and introduced this plasmid into the tr-SCV where it restored the growth of the tr-SCV to wild-type levels (Fig. 1f).

**Fig. 2.**
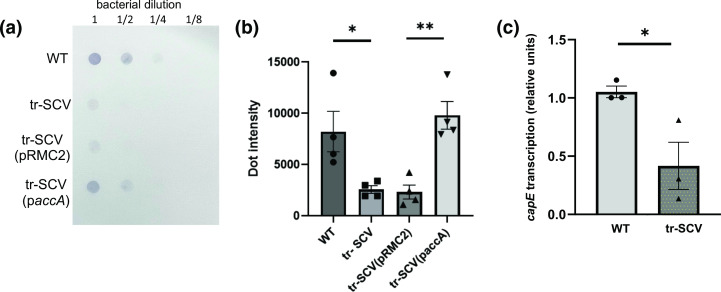
The tr-SCV of *S. aureus* produces less capsule. (**a**) Capsule immunoblots of the wild-type (WT) *S. aureus* strain Newman, the triclosan-resistant SCV (tr-SCV), the triclosan-resistant SCV containing the empty complementing pRMC2 plasmid and the triclosan-resistant SCV containing the complementing p*accA* plasmid. (**b**) Densitometric analysis of replicate immunoblots quantifying capsule production at an OD_600_ of 2. (**c**) Transcription of the *capE* gene was quantified by qRT-PCR in both the wild-type and tr-SCV of strain Newman. Statistics were performed using a paired *t*-test where * indicates significance of *P*<0.05 and ** indicates significance of *P*<0.01.

To examine whether capsule production is affected in the tr-SCV we performed immunoblots with capsule antiserum. Relative to the wild-type Newman strain, the tr-SCV produced significantly less capsule, an effect that was complemented by expressing the *accA* gene from the pRMC2 plasmid (Fig. 2a, b) [25]. To determine whether this effect on capsule production was at the transcriptional level, qRT-PCR was used to quantify the level of expression of the *capE* gene, where the tr-SCV expressed significantly less of this gene (Fig. 2c).

**Fig. 3.**
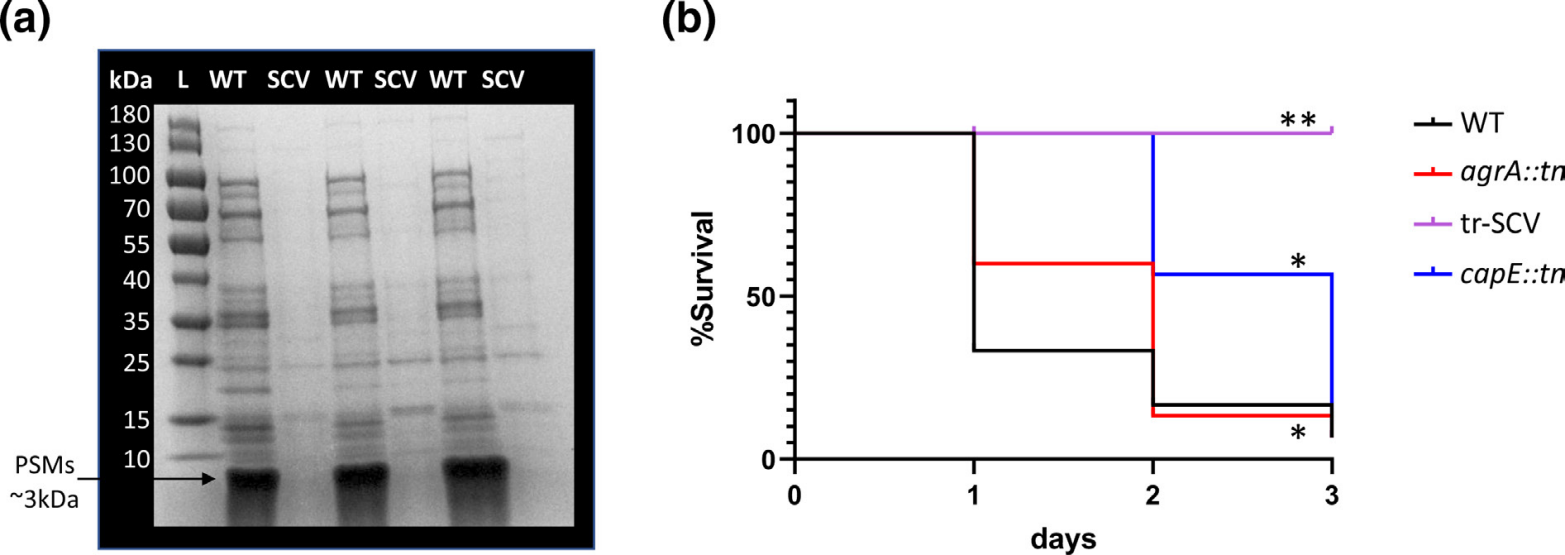
The tr-SCV produces less PSMs and is attenuated *in vivo*. (**a**) There was no detectable PSM band in the SCV supernatant when compared to that in the supernatant of the wild-type Newman strain (WT). (**b**) The tr-SCV is significantly attenuated relative to the wild-type strain, an isogenic *capE* mutant (*capE::tn*), and an isogenic *agrA* mutant of Newman (*agrA::tn*) in a *G. mellonella* model of infection. Statistics were performed using log-rank (Mantel–Cox) tests, and all three mutants were significantly less virulent when compared to the wild-type Newman strain. * indicates a *P*-value of <0.001 and ** indicates a *P*-value of <0.0001.

A common feature of *S. aureus* SCVs is that they do not produce cytolytic toxins. We examined this for the tr-SCV by comparing the quantity of phenol-soluble modulins (PSMs) they produce with that of the wild-type strain. PSMs are small surfactant-like molecules that include the delta toxin, the major cytolytic component encoded by the virulence regulating Agr system. Both the wild-type and tr-SCV were grown for 18 h in TSB, and the proteins in the supernatant were harvested and examined on an SDS-PAGE gel, were the PSMs migrate together as a 3 kDa band ahead of the sample dye front [33]. There was a robust PSM band in the supernatant of Newman, but none was visible in the tr-SCV supernatant (Fig. 3a). Given the pleiotropic effects of the tr-SCV-associated mutation on virulence phenotypes, we examined whether it would be attenuated in an insect model of virulence. *Galleria melonella* is an insect model that has been used previously to examine tr-SCV virulence and as such we used it here to examine whether our tr-SCV (with a mutated *accA* gene) was attenuated [15, 32]. *G. mellonella* larvae were injected with 10 µl of 1×10^8^ c.f.u. of the wild-type or tr-SCV strains, alongside a capsule (*capE*) and an Agr (*agrA*) mutant to examine the relative effect the loss of either capsule alone (*capE* mutant) or toxins and capsule (*agrA* mutant) has on virulence in this model. Of the mutants tested, the tr-SCV was the most attenuated, killing no larvae over the 3-day period of the experiment (Fig. 3b). Both the *capE* and *agrA* mutants were also attenuated relative to the wild-type strains, but not to the same degree as the tr-SCV (Fig. 3b, *P*<0.001).

In previous work we analysed capsule production by both *hemB* and *menD* SCVs of *S. aureus *, where we found that those that were auxotrophic for menadione (*menD*) produced little capsule, whereas those that were auxotrophic for hemin were unaffected [20]. In this study we demonstrate that SCV associated with triclosan resistance and auxotrophic for fatty acids also produce less capsule, an effect that appears to be mediated at the transcriptional level. We also demonstrate that tr-SCV is significantly attenuated for virulence in an insect model of infection. This is likely due to the reduced capsule and PSM production by this variant, but given that it was more attenuated that both a *capE* and an *agrA* mutant, the slow growth rate and associated metabolic changes [15–19] are also likely to contribute to the reduced virulence of this tr-SCV.

Triclosan is an antimicrobial chemical that is used in a wide array of applications, ranging from its use in cosmetics and toothpastes to prevent infections, through the decolonization of MRSA patients, to being embedded in sutures for the closure of post-operative wounds. However, this use has been significantly reduced in both the USA and Europe due to concerns over the emergence of resistant bacterial strains. In this study we add to the literature surrounding triclosan resistance, and demonstrate that in addition to published effects on toxin production, tr-SCVs also do not produce capsule and are attenuated in an insect model of virulence. Given the effect that the associated mutations have on their potential pathogenicity, it is unlikely that tr-SCVs will impact directly on patient outcome. However, given the perception that SCVs are frequently misdiagnosed clinically, alongside their ability to persist in harsh environments and revert to the wild-type growth rates, it is an aspect of the biology of *S. aureus* that is worth monitoring.
