# Corrigendum: Triclosan-resistant small-colony variants of *Staphylococcus aureus* produce less capsule, less phenol-soluble modulins, and are attenuated in a *Galleria mellonella* model of infection

**DOI:** 10.1099/mic.0.001419

**Published:** 2023-11-27

**Authors:** Dina Altwiley, Tarcisio Brignoli, Seána Duggan, Ruth C. Massey

**Affiliations:** ^1^​ School of Cellular and Molecular Medicine, University of Bristol, Bristol, UK; ^2^​ Department of Biological Sciences, University of Jeddah, Jeddah, Saudi Arabia; ^3^​ Dipartimento di Bioscienze, Università degli studi di Milano, Milan, Italy; ^4^​ MRC Centre for Medical Mycology, University of Exeter, Exeter, UK; ^5^​ Schools of Microbiology and Medicine, and the APC Microbiome Ireland, UCC, Cork, Ireland

## Full-Text

In the published version of this article, there was an omission in the Funding Information.

The BBSRC funder grant reference number, BB/R009724/1, should have been included.

Previously, the Funding Information appeared as below:

This work was funded by a Wellcome Trust funded Investigator award to R.C.M. (grant reference number: 212258/Z/18/Z), and a grant from the BBSRC. D.A. is funded by a PhD studentship awarded by the Saudi Arabian Cultural Bureau.

The correct Funding Information should have appeared as:

This work was funded by a Wellcome Trust funded Investigator award to R.C.M. (grant reference number: 212258/Z/18/Z), and a grant from the BBSRC (grant reference number: BB/R009724/1) . D.A. is funded by a PhD studentship awarded by the Saudi Arabian Cultural Bureau.

The author apologizes for any inconvenience caused.

